# The Cyanophage Molecular Mixing Bowl of Photosynthesis Genes

**DOI:** 10.1371/journal.pbio.0040264

**Published:** 2006-07-04

**Authors:** Emma Hill

Among the wealth of microbial organisms inhabiting marine environments, cyanobacteria (blue-green algae) are the most abundant photosynthetic cells. *Prochlorococcus* and *Synechococcus*, the two most common cyanobacteria, account for 30% of global carbon fixation (through the photosynthetic process in which sugars are manufactured from carbon dioxide and water). By drawing on natural resources, these microbes use photosystems (PS) I and II (the two reaction centers in photosynthesis) to harness energy.

Intriguingly, some viruses that infect cyanobacteria (called cyanophage), carry genes that encode two PSII core reaction-center proteins: PsbA (the most rapidly turned over core protein in all oxygen-yielding photosynthetic organisms) and PsbD (which forms a complex with PsbA). By expressing their own copies of *psbA* and *psbD* during infection, these cyanophages have managed to co-opt host genes to suit their own purposes: enhancing photosynthesis. It seems likely that they do this in the interests of their own fitness, since cyanophage production is optimal when photosynthesis is maintained during infection.

Until recently, only a small sample of cyanophages had been examined, leaving open the questions of how widespread PSII genes are in these organisms and where the genes came from. To answer these questions, Matthew Sullivan, Debbie Lindell, Sallie Chisholm, and colleagues examined a pool of 33 cyanophage isolates (cultured from samples collected from the Sargasso Sea and the Red Sea), along with data already available for nine other cyanophages, for the presence of *psbA* and *psbD* genes. They found *psbA* was present in 88% and *psbD* in 50% of the cyanophages studied. By analyzing the sequences of these genes along with those from *Prochlorococcus* and *Synechococcus* host genes, they reconstructed the evolutionary history of how the PSII genes entered the phage genomes.

Cyanophages are divided morphologically into three main families (Podoviridae, Myoviridae, and Siphoviridae). Looking at the distributions of the PSII genes across the different families, Sullivan, Lindell, et al. saw that *psbA* was present in all myoviruses and all *Prochlorococcus* podoviruses, but not in *Prochlorococcus* siphoviruses or *Synechococcus* podoviruses. The high levels of sequence conservation between the different cyanophages suggest that this gene is probably functional and that it is likely to increase the reproductive fitness of the phage. The length of the latent period may impact the distribution pattern of *psbA* among these phage groups. However, more information about the physiological characteristics of cyanophages is needed to further investigate these possibilities.

The second gene, *psbD*, was less prolific but was seen in four of the 20 *Prochlorococcus* myoviruses and 17 of the 20 *Synechococcus* myoviruses examined—all of which also encoded *psbA*. Myoviruses are known to infect a wider range of cyanobacteria than the other cyanophage families. Indeed, when investigated, the *psbD*-encoding myoviruses correlated with those known to have a broader host range. Perhaps the co-opting of both PSII genes ensures a functional PsbA–PsbD protein complex to enhance infection for these cyanophages that are able to infect a wider range of hosts.

To determine when the PSII genes had been transferred into the phage and from where, Sullivan, Lindell, et al. investigated the nucleotide sequences of *psbA* and *psbD* from both *Prochlorococcus* and *Synechococcus* host and cyanophage. Using meticulous sequence analyses and standard statistical methods, they generated phylogenetic trees to explain the evolutionary history of these two PSII genes.

By analyzing the clusters of sequence types within the resulting tree, the authors saw evidence that *psbA* was transferred from the cyanobacteria host genome into the phage genome on four independent occasions and two separate occasions for *psbD*. Exchange events were generally host-range specific, meaning that *Prochlorococcus* genes transferred to *Prochlorococcus* phages, and so on. However, a few intriguing exceptions, where genes did not cluster with their hosts, were observed; these might result from genetic exchange between members of two different phage families (one of broader host range) during co-infection of the same host.

Sullivan, Lindell, et al. were also able to use their dataset to investigate a previous suggestion that alterations in the nucleotide distributions within individual PSII genes (creating a kind of patchwork gene) demonstrate that intragenic recombination has taken place. Indeed, they confirm that this occurs among *Synechococcus* myoviruses and *Prochlorococcus* podoviruses. In some cases involving *Synechococcus*, intragenic recombination appears to have happened in both host-to-phage and phage-to-host directions for both genes; and, for some *Prochlorococcus* genes, DNA from an unknown source also seems to have been inserted. Occasionally, intragenic exchanges are also seen between *Synechococcus* hosts.

The authors compare their cultured results to those from wild phage sequences from the Pacific Ocean and see that much of the natural diversity is similar to the sequences from the cyanophage isolates, despite their origination from different ocean basins. Overall, therefore, a considerable amount of genetic shuffling takes place within these two PSII genes in cyanophages, and this creates a reservoir of photosynthetic diversity from which both host and phage are likely to benefit. This study offers a compelling example of global-scale microbial and phage co-evolution that likely influences the biological success of these prolific marine organisms.

## 

**Figure:**
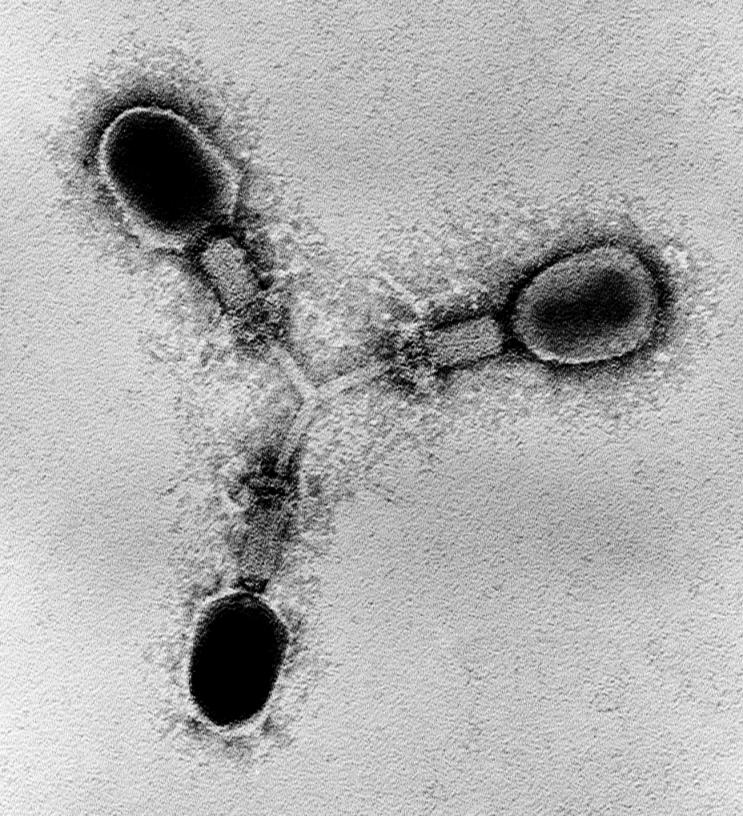
Cyanophages–viruses that infect photosynthetic marine bacteria–not only possess genes for photosynthesis but also exchange genetic material with their cyanobacterial hosts.

